# Behavioral adaptation of sympatric rodents to early germination of oak acorns: radicle pruning and embryo excision

**DOI:** 10.3389/fpls.2023.1135312

**Published:** 2023-05-09

**Authors:** Mingming Zhang, Xifu Yang, Zhong Dong, Shuyuan Liu, Huanhuan Chen, Xianfeng Yi

**Affiliations:** ^1^ College of Agriculture, Henan University of Science and Technology, Luoyang, China; ^2^ State Key Laboratory of Integrated Management of Pest Insects and Rodents, Institute of Zoology, Chinese Academy of Sciences, Beijing, China; ^3^ College of Food and Bioengineering, Henan University of Science and Technology, Luoyang, China; ^4^ School of Life Sciences, Qufu Normal University, Qufu, China

**Keywords:** radicle pruning, embryo excision, seed germination, seed dispersal, plant-animal interactions

## Abstract

The seed germination schedule is a key factor affecting the food-hoarding behavior of animals and the seedling regeneration of plants. However, little is known about the behavioral adaptation of rodents to the rapid germination of acorns. In this study, we provided *Quercus variabilis* acorns to several rodent species to investigate how food-hoarding animals respond to seed germination. We found that only *Apodemus peninsulae* adopted embryo excision behavior to counteract seed germination, which is the first report of embryo excision in nonsquirrel rodents. We speculated that this species may be at an early stage of the evolutionary response to seed perishability in rodents, given the low rate of embryo excision in this species. On the contrary, all rodent species preferred to prune the radicles of germinating acorns before caching, suggesting that radicle pruning is a stable and more general foraging behavior strategy for food-hoarding rodents. Furthermore, scatter-hoarding rodents preferred to scatter-hoard and prune more germinating acorns, whereas they consumed more nongerminating acorns. Acorns with embryos excised rather than radicles pruned were much less likely to germinate than intact acorns, suggesting a behavioral adaptation strategy by rodents to the rapid germination of recalcitrant seeds. This study provides insight into the impact of early seed germination on plant–animal interactions.

## Introduction

Antagonism and/or cooperation between rodents and plant seeds can regulate the ecological and evolutionary processes and maintain ecosystem stability and complexity (Zhang and Yi, 2021; Zhang et al., 2021; Yang et al., 2022; Yu et al., 2022; Liu et al., 2022). Food-hoarding rodents act as both seed dispersers and seed predators of many plant species, and this interaction is often associated with specific seed traits (Vander Wall and Beck, 2012; Rusch et al., 2013). Seed traits (such as seed size/mass, coat thickness, and tannin) have been shown to influence seed selection, scatter-hoarding, and seed dispersal by seed-eating animals (Cao et al., 2016; Dylewski et al., 2020; Chen et al., 2022; Yang et al., 2023). Among them, seed perishability (due to germination schedules) exerts a particularly prominent effect on the outcome of hoarder–plant interactions (Smallwood and Peters, 1986; Hadj-Chikh et al., 1996; Yi et al., 2012a; Xiao et al., 2022). In addition, rodent species with different morphological and behavioral characteristics, such as body mass and personality, usually show different roles in the interaction between rodents and plant seeds (Siepielski and Benkman, 2008; Lichti et al., 2017; Brehm and Mortelliti, 2022).

Many seed hoarders, particularly rodents, exhibit several behavioral responses that reduce seed perishability and cache loss (Fox, 1982; Xiao et al., 2013; Mittelman et al., 2021). Specific strategies used by rodents include radicle pruning or embryo excision, which are thought to reduce seed perishability and aid in seed cache management (Jansen et al., 2006; Steele et al., 2006; Cao et al., 2011). However, in response to many of these strategies, plants appear to have countered such rodent responses with their own adaptive strategies for survival and regeneration (Vander Wall, 2010), including rapid regeneration (Cao et al., 2011), production of multiple, deeper, or atypical embryos (Mceuen and Steele, 2005; Zhang et al., 2014a), altered radicle morphology (e.g., pronounced cotyledonary petiole), and enhanced tolerance to radicle and acorn pruning (Vallejo-Marin et al., 2006; Yi et al., 2012b). Recent studies suggest that the mutual benefit efficiency of both plants and animals may be improved in an arms race between rodent and plant seed predation and antipredation (Cao et al., 2011; Cao et al., 2022).

Unlike the dormant acorns of red oaks (RO, subgenus *Erythrobalanus*) and Qinggang oaks (QG, subgenus *Cyclobalanopsis*), the nondormant acorns of white oak species (WO, subgenus *Quercus*) produce recalcitrant acorns that exhibit early germination after seed fall or even while still attached to the tree in autumn (Fox, 1982; Steele et al., 2006). This rapid germination schedule of white oaks, which results in the rapid transfer of energy from the acorn to an inedible, cellulose-rich tap root, is widely interpreted as an adaptive strategy to counter intense seed predation by rodents (Fox, 1982; Hadj-Chikh et al., 1996). These robust and rapidly developing taproots of white oak species also show a high capacity to regenerate into normal seedlings even when rodents damage or remove acorns (Yi et al., 2013; Yi et al., 2019). It appears that squirrels in particular have developed a rather specific strategy for dealing with the early germination in white oaks. Several members of the genus *Sciurus* in North America (*Sciurus carolinensis*, *S. aureogaster*, *S. niger*) and others belonging to three genera (*Sciurotamias davidianus*, *Callosciurus erythraeus*, and *Dremomys rufigenis*) from southeast Asia have been reported to excise the embryo of white oak acorns (e.g., *Quercus alba*, *Q. variabilis*, *Q. aliena* var. *acutiserrata*, and *Q. serrata* var. *brevipetiolata*) prior to storage (Fox, 1982; Xiao et al., 2009; Xiao et al., 2010; Xiao et al., 2013). This specific behavior of embryo excision permanently arrests germination and functionally kills developing acorns but allows long-term storage of the cotyledon for up to 6 months (Xiao and Zhang, 2012; Yi and Liu, 2014). Moreover, an innate basis for embryo excision behavior has been reported in eastern grey squirrels in the USA (Steele et al., 2006) and a population of red-cheeked squirrels in China (Xiao and Zhang, 2012).

It is especially compelling that many other food-hoarding rodents face the same challenges of early WO germination (Yang et al., 2012; Yi et al., 2012b). In North America, for example, several rodent species (including southern flying squirrels (*Glaucomys volans*), eastern chipmunks (*Tamias striatus*), and white-footed mice (*Permomycus leucopus*)) selectively cache RO acorns over WO (Steele et al., 2006). Likewise, these and other species in both North America and Asia rely on other strategies to manipulate scatter hordes of early germinating seeds, such as pruning acorns from developing taproots or repeated pruning of taproots or epicotyls (Jansen et al., 2006; Cao et al., 2011; Yi et al., 2021). Therefore, it is not clear whether sympatric rodents adopt different foraging strategies for seed germination.

In this study, we provided both germinating and nongerminating acorns of *Q. variabilis* (an early germinating oak species) to individual field food-hoarding rodents, including scatter hoarders and larder hoarders, with the former potentially having a greater impact on seedling establishment. We predicted that (1) there will be differences in acorn consumption and scatter-hoarding decisions of rodents with different morphological and behavioral characteristics, because differentiation in hoarding behavior may help sympatric species to harvest more energy, which contributes to their coexistence in the field (Cheng et al., 2005; Meng et al., 2022); (2) rodents will remove the radicle or embryo of germinating acorns before hoarding, thereby reducing seed perishability in caches (Steele et al., 2006; Yi et al., 2012a); and (3) embryo-excised acorns are far less likely to germinate than those with radicles pruned or intact ones because the embryo is a necessary condition for maintaining seed germination (Yi et al., 2013), and radicle-pruned acorns successfully regerminated when the radicle was unlignified (Zhang et al., 2016).

## Materials and methods

### Study site

The study was carried out in central China in the Tianchishan Mountains (1,200 m above sea level, 33° 45′–33° 85′ N, 111° 75′–112° 45′ E), nested in the Funiushan Mountain systems of central China, located in a transition belt between the subtropical and northern warm zones and also on the dividing line between the eastern boundary and the ancient northern boundary of China’s animal division. The annual mean temperature of the region is 12.4°C, with a maximum monthly average temperature of 24.4°C in July and a minimum of −1°C in January, and the annual mean rainfall is 647.6 mm, with a maximum of 136.6 mm in July and a minimum of 6.1 mm in January. The frost-free period is 200 days/year. The vegetation is dominated by deciduous trees of *Q. variabilis*, *Q. aliena*, and *Q. aliena* var. *acutiserrata* secondary forests, mixed with other broad-leaved tree species, e.g., *Q. serreta*, *Castanea mollissima*, *C. seguinii*, and *Diospyros kaki*. The shrub understorey is diverse and rich in endemic taxa (e.g., *Corylus* spp.), the litter is abundant with an average thickness of 3–5 cm, and the soil is mainly montane brown soil. The main seed predators are *A. peninsulae*, *Cansumys canus*, *Niviventer confucianus*, *S. davidianus*, *Garrulus glandarius*, *Sus scrofa*, and rare *Callosciurus erythraeus* (Yi and Wang, 2015).

### Rodent species

Steel-framed live traps (H × W × L: 9 cm × 10 cm × 25 cm), baited with peanuts and carrots, were placed in the forest at 5-m intervals along four transects at 0900 hours. The live traps were checked twice a day, in the early morning and late afternoon, to ensure the safety of the captured rodents. Animals captured during each visit were immediately transported by vehicle to the laboratory animal facility within 30 min. Trapping was stopped on days with bad weather, i.e., heavy rain. All trapping was conducted at the end of the breeding season, and none of the rodents appeared to be in breeding condition. Healthy and mature rodents were transported to the laboratory and individually housed in steel frame cages (H × W × L: 40 cm × 50 cm × 90 cm) under natural temperature (15°C–25°C) and photoperiod (14 h of light) (Zhang et al., 2013a). All animals received carrots, peanuts, tree seeds, and water *ad libitum*. To reduce the stress response of rodents, before the seed dispersal experiment in the enclosure, rodents captured in the field were reared for a period of time and fed experimental seeds 1 week before the formal experiment to allow them to adapt to the environment and seeds. After the enclosure experiment, all rodents were released at the original capture sites in the forest. No animal died during the capture and laboratory feeding process. In this study, we captured all common rodents, including *A. peninsulae*, *S. davidianus*, *N. confucianus*, and *C. canus*, in the Tianchishan Mountains. However, a rarely distributed species, *Tamias sibiricus*, was captured in the Dailing district, Heilongjiang Province, and transported to the study area.

### Acorn selection and marking

Healthy acorns of *Q. variabilis* of uniform size (4.6 ± 0.06 g, dominant tree species in Tianchi Mountain) were selectively collected from more than 20 trees using seed traps. Acorns were labeled with plastic tags according to the previously published method (Zhang et al., 2013a). Each acorn was drilled with a 0.3-mm-diameter hole at the base and then attached with a writable plastic tag (2.5 cm × 3.5 cm, <0.3 g) using a thin 10-cm steel thread. Each tag was individually coded so that all acorns could be easily relocated and identified. If acorns were buried in soil or leaf litter by rodents, attached tags were often left on the ground surface to facilitate our location.

### Experiments in semi-natural enclosures

To determine the consumption and scatter-hoarding preferences of germinating and nongerminating acorns in the five rodent species, we conducted experiments in semi-natural enclosures previously established in an open, nonforested area. A seed station was set in the center of each enclosure to release acorns, and a rodent nest was set up in one corner. Each enclosure was covered at the top with plastic netting to prevent predators from entering. Each rodent was released into an enclosure between 1330 and 1400 hours on the day of the experiment. Within the enclosures (5 m × 5 m), we provided 10 pairs of germinating (with radicles 0.3–0.5 cm) and nongerminating *Q. varialibis* acorns (**Supplementary Figure S1**) to each individual of the four rodent species, *A. peninsulae*, *N. confucianus*, *C. canus*, *T. sibiricus*, and 20 pairs to *S. davidianus*, with the placement of each individual seed randomized. The acorns were then relocated and examined the following day at 1100 hours. In total, we tested 21 A*. peninsulae* (8♀, 18.5 ± 3.9 g; 13♂, 22.3 ± 8.3 g), 8 *S. davidianus* (2♀, 209.5 ± 14.1 g; 6♂, 223.6 ± 8.2 g), 14 *N. confucianus* (3♀, 66.0 ± 4.6 g; 11♂, 64.5 ± 3.4 g), 8 *C. canus* (2♀, 74.6 ± 4.2 g; 6♂, 78.4 ± 2.3 g), and 8 *T. sibiricus* (5♀, 87.1 ± 3.4 g; 3♂, 112.6 ± 7.9 g).

Following Wang et al. (2020), seed fates were defined as removal (acorns removed from the seed station by rodents), consumption (including acorns eaten both *in situ* and after removal, and acorns taken to underground burrows, which were more likely to be eaten eventually and had little significance for the renewal of seedlings), and scatter-hoarding (acorns removed from the seed station and buried by rodents under leaf litter or in the soil, with each cache containing one or a few acorns, and most caches containing only one acorn), with removal including both consumption and scatter-hoarding. Once we observed that a rodent had scatter-hoarded the seeds, we classified it as a scatter hoarder; otherwise, it was considered a larder hoarder. We also recorded the number of acorns removed and buried to see which type of acorn (germinating or nongerminating) was preferred by the rodents. The germinating acorns in the caches were carefully checked to determine if the radicles or embryos had been pruned or removed by rodents. Acorns with radicles pruned or embryo excised were harvested and stored at 4°C for later germination experiments.

### Acorn germination experiments

To investigate the effect of radicle pruning or embryo excision by 21 A*. peninsulae* on germination rates, we sowed acorns with radicle pruning or embryo excision paired with an intact acorn in organic composite soil filled into plastic trays arranged in a 5 × 10 grid. After planting the acorns in plastic trays, we randomly covered a thin layer of dead branches and fallen leaves to simulate natural conditions. In total, we collected and planted 24 embryo-excised, 50 radical-pruned, and 75 intact acorns of *Q. variabilis*. The plant containers were protected by a metal wire mesh (1.5 cm ×1.5 cm) and kept on the forest floor in the Tianchishan Mountains. The mesh was regularly cleared of fallen leaves to avoid affecting the growth of germinating seedlings and reduce the impact on container water and temperature. Germination rates were measured 180 days after cultivation.

### Ethical statement

The ethical treatment of animals, including live trapping, handling, housing, and behavioral procedures, was approved by Henan University of Science and Technology, where the studies were conducted. No animal died during capture and behavioral experiments.

### Data analysis

Generalized linear mixed models (GLMMs) were used to analyze differences in seed removal, consumption (acorns eaten or carried into underground burrows), and scatter-hoarding between rodent species and rodent individuals as random effects for data from enclosures (**Supplementary Table S1**). Tuckey’s test (implemented through the emmeans package) was applied for *post-hoc*, pairwise comparison of seed fates and different rodent groups. Analyses of variance (ANOVAs) with the Wald Chi-square test were performed to test the significance of the fixed categorical variables in the GLMMs using the ANOVA function in the car package (Fox and Weisberg, 2019). GLMMs using the glmer function in the lme4 package (Bates et al., 2015) were applied with seed fates (that is, removal, consumption, and scatter-hoarding of acorns) as response variables and the germinating states (that is, germinating versus nongerminating) of acorns as independent variables, with rodent individuals as random effects for enclosure data (**Supplementary Table S2**). Pearson correlation analysis was used for the difference between proportion of radicle pruning (including embryo excision) and the proportion of scatter-hoarding of germinating acorns. Chi-square tests were used to test the difference in germination rates between embryo excision, radicle pruning, and intact acorns (**Supplementary Table S3**). Data analyses were performed using the base, stats, lme4, car, and emmeans packages in R software (4.2.1) (R Core Team, 2022).

## Results

### The general pattern of five rodent foraging preferences

**Figure 1:**
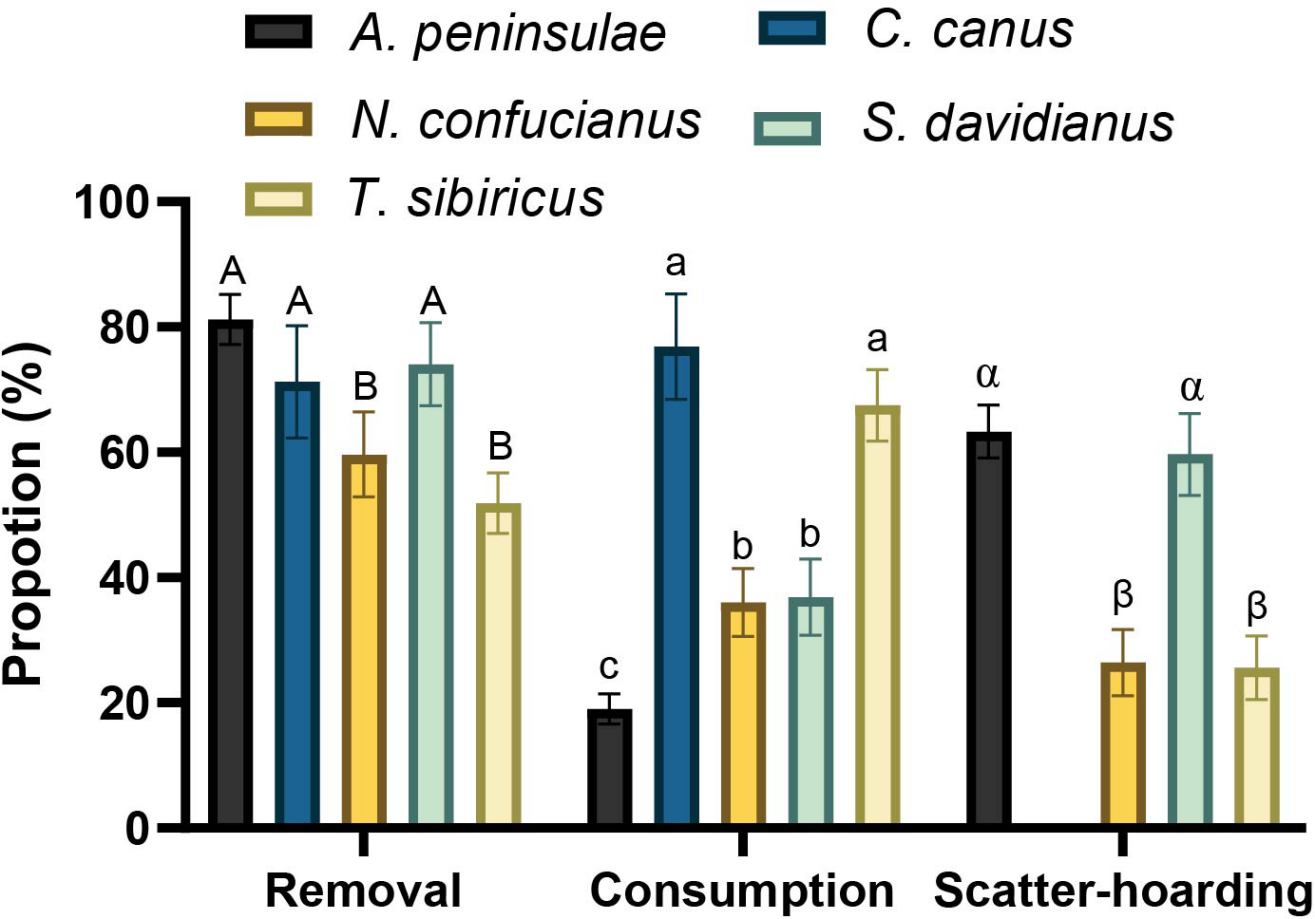
Proportions of *Q. variabilis* acorns that were removed, consumed (acorns that were eaten or taken to an underground burrow), and scatter-hoarded by five rodent species. Data are expressed as mean ± SE. Different letters denote significant differences between rodent species (Tukey’s test; *p* < 0.05; **Supplementary Table S1**).

In total, 82.4%, 76.9%, 62.5%, 96.6%, and 93.1% of *Q. variabilis* acorns were harvested (eaten or removed) by *A. peninsulae*, *C. canus*, *N. confucianus*, *S. davidianus*, and *T. sibiricus*, respectively. The proportions of the *Q. variabilis* acorns removed (*χ* ^2^ = 53.172, *df* = 4, *p* < 0.001), consumed (acorns that were eaten or carried into underground burrows; *χ* ^2^ = 186.54, *df* = 4, *p* < 0.001), and scatter-hoarded (*χ* ^2^ = 122.77, *df* = 3, *p* < 0.001) differed significantly between the five species of rodents (**Figure 1**). We showed that larder hoarder of *C. canus* and scatter hoarder of *T. sibiricus* consumed more *Q. variabilis* acorns than the other three rodent species; *A. peninsulae* consumed less than 20%. However, *A. peninsulae* and *S. davidianus* scatter-hoarded more acorns than the other three rodent species in this study (**Figure 1**; **Supplementary Table S1).**

### Selection preferences for paired germinating and nongerminating acorns

**Figure 2:**
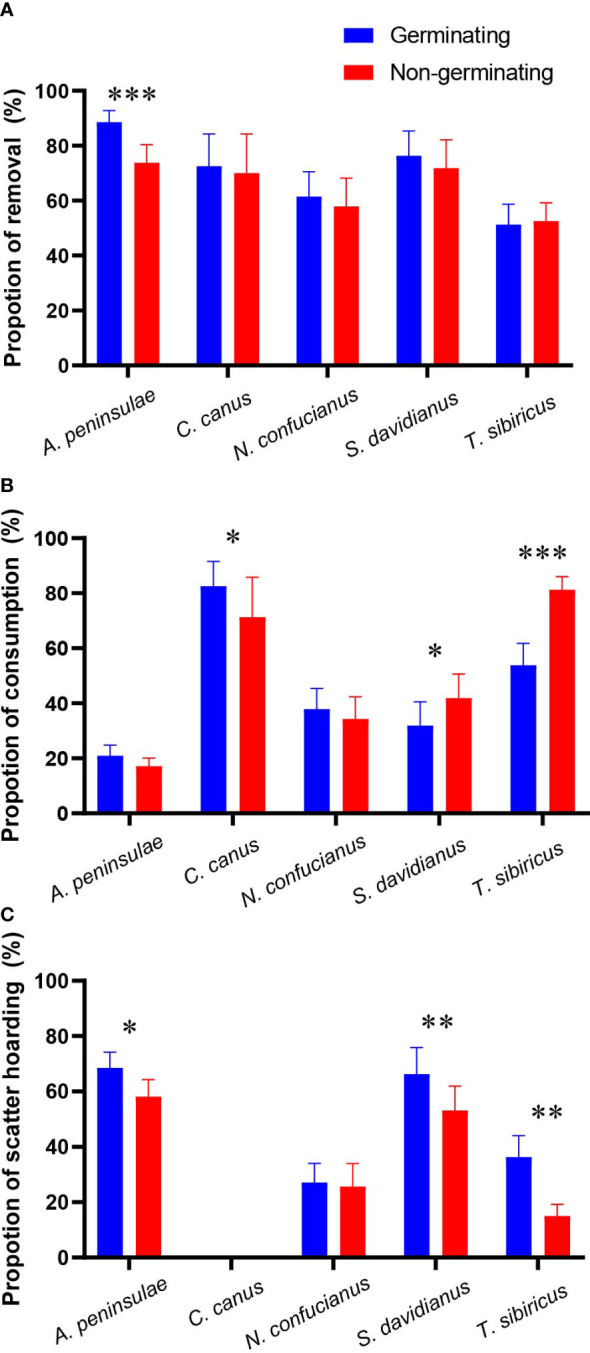
Preferences of the five rodent species in removing **(A)**, consuming (**B**, acorns that were eaten or taken to an underground burrow), and scatter-hoarding **(C)** germinating and nongerminating acorns of *Q. variabilis*. Data are expressed as mean ± SE. ^*^ *p* < 0.05; ^**^ *p* < 0.01; ^***^ *p* < 0.001 (**Supplementary Table S2**).

We showed that the five rodent species did not discriminate nongerminating acorns against germinating ones in the paired experiments except *A. peninsulae* which removed more germinating acorns than nongerminating acorns (*z* = 4.432, *p* < 0.001; **Figure 2A**; **Table 1**; **Supplementary Table S2**).

**Table 1:** Summary of significant differences in seed fates (acorn removal, consumption, and scatter-hoarding) between germinating and nongerminating acorns by different rodent species based on generalized linear mixed models.

Seed fates	Rodent species		Estimate	Std. error	*z*	*p*-value
Acorn removal	*A. peninsulae*	(Intercept)	3.465	0.663	5.229	<0.001
		Nongerminating	−1.464	0.330	−4.432	<0.001
Acorn consumption	*C. canus*	(Intercept)	9.406	3.591	2.619	0.009
		Nongerminating	−1.464	0.605	−2.420	0.016
	*S. davidianus*	(Intercept)	−0.861	0.430	−2.001	0.045
		Nongerminating	0.536	0.260	2.063	0.039
	*T. sibiricus*	(Intercept)	0.161	0.292	0.550	0.582
		Nongerminating	1.383	0.376	3.675	<0.001
Acorn scatter-hoarding	*A. peninsulae*	(Intercept)	0.964	0.306	3.149	0.002
		Nongerminating	−0.578	0.230	−2.518	0.012
	*S. davidianus*	(Intercept)	0.690	0.492	1.401	0.161
		Nongerminating	−0.698	0.260	−2.679	0.007
	*T. sibiricus*	(Intercept)	−0.610	0.323	−1.888	0.059
		Nongerminating	−1.248	0.404	−3.086	0.002

In our study, *T. sibiricus* and *S. davidianus* preferred to consume nongerminating acorns, while *C. canus* consumed significantly fewer nongerminating acorns. However, *A. peninsulae* and *N. confucianus* showed no preference over germinating and nongerminating acorns (**Figure 2B**; **Table 1**; and **Supplementary Table S2**). The scatter-hoarding species *T. sibiricus*, *S. davidianus*, and *A. peninsulae* cached more germinating acorns than nongerminating ones (**Figure 2C**; **Table 1**; **Supplementary Table S2**).

### Radicle pruning preferences of rodents

**Figure 3:**
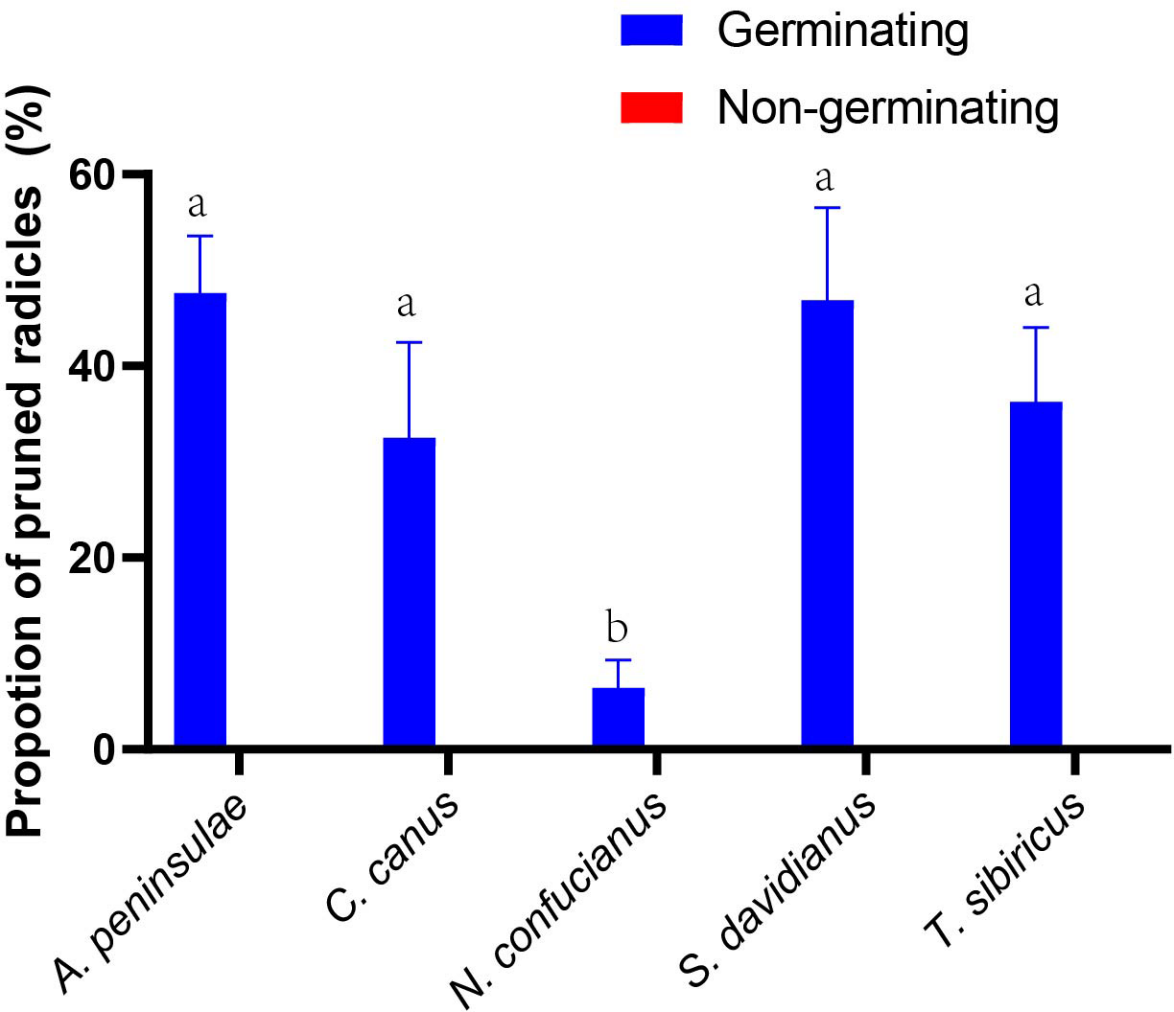
Preferences for radicle pruning (including embryo excision by *A. peninsulae*) of germinating and nongerminating acorns of *Q. variablilis* by the five rodent species. The data are expressed as mean ± SE. Different letters denote significant differences between rodent species (Tukey’s test; *p* < 0.05; **Supplementary Table S1**).

**Figure 4:**
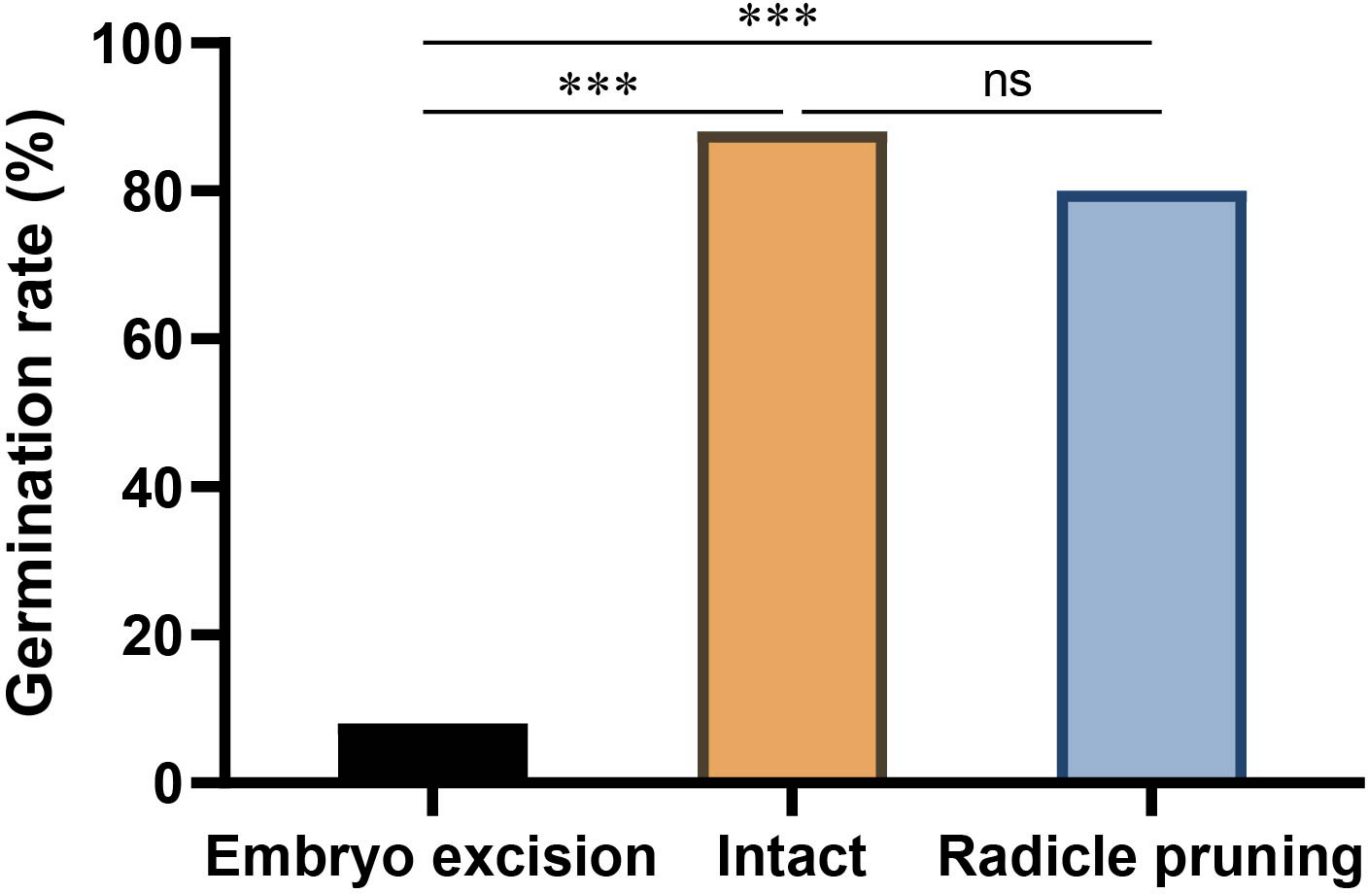
Effects of embryo excision and radicle pruning on acorn germination of *Q. variabilis.* The data are expressed as mean ± SE. ^***^ *p* < 0.001; ns, nonsignificant difference (**Supplementary Table S3**).

Rodents with a strong scatter-hoarding ability pruned a higher proportion of germinating acorns than those exhibiting a weaker scatter-hoarding ability (**Figure 3**; **Supplementary Table S1**). There was a significant positive correlation between the proportion of radicle pruning (including embryo excision) and the proportion of scatter-hoarding of germinating acorns (*r* = 0.818, *n* = 59; *p* < 0.001). The larder hoarder of *C. canus* pruned the acorns’ radicles up to 32.5 ± 9.96% (Mean ± SE; **Figure 3**). More importantly, we first showed that 8.09% of the *Q. variabilis* acorns were embryo excised by *A. peninsulae* (**Figure 4**; **Supplementary Table S3**).

### Effects of embryo excision and radicle pruning on acorn germination

In the enclosure, 8.09% and 39.5% of *Q. variabilis* acorns had embryos excised and radicle pruned by *A. peninsulae*, respectively. Our germination experiments showed that 8% and 80% of the *Q. variabilis* acorns with embryos excised and radicles pruned successfully germinated. Embryo excision by *A. peninsulae* significantly stopped acorn germination of *Q. variabilis* (*χ* ^2^ = 51.532, *df* = 1, *p* < 0.001), but acorn germination was not significantly inhibited by radicle pruning (**Figure 4**; **Supplementary Table S3**).

## Discussion

### Hoarding strategies of the sympatric rodent species

Sympatric rodents often have different foraging strategies due to competition for food resources (Lu and Zhang, 2008; Zhang et al., 2014b; Meng et al., 2022). Our findings presented further evidence for these observations (Zhang et al., 2013a; Zhang et al., 2014a; Gu et al., 2021). In our study, *C. canus* is the largest solitary animal among nocturnal rodents with strong territorial behavior and a strong ability to defend the nest (Zhang’s personal observations), so larder-hoarding has been its efficient food-hoarding strategy (Zhang et al., 2022). With the exception of *C. canus*, other rodents had varying degrees of scatter-hoarding ability. We found that small-sized *A. peninsulae* (20.8 ± 0.7 g) and the large-sized *S. davidianus* (220.5 ± 6.9 g) removed and scatter-hoarded more acorns, suggesting that rodent body mass is not a stabilizing factor affecting their food-hoarding strategies (Munoz and Bonal, 2008; Zhang et al., 2022). *Apodemus peninsulae* consumed fewer acorns because body mass has a positive effect on rodent consumption (Cheng et al., 2005). However, *T. sibiricus* consumed more acorns than *S. davidianus*, possibly because *T. sibiricus* was not a natively trapped animal and rodents preferred to consume seeds when encountering new environments and food resources. Consistent with many previous studies, our results suggest that different rodents have different implications for forest regeneration, as scatter-hoarding favors oak seed dispersal (Chang and Zhang, 2011; Vander Wall and Beck, 2012; Rusch et al., 2013). In addition, it is worth noting that *N. confucianus* in our study exhibits scatter-hoarding ability. This observation is inconsistent with studies conducted in the Donglingshan area of the Palearctic region, where *N. confucianus* mainly larder hoards food (Lu and Zhang, 2008; Zhang et al., 2017), but consistent with studies conducted in the Xishuangbanna and Dujiangyan areas of the Eastern Boundary and the central subtropics, where the same species both scatter and larder hoards food (Zhang et al., 2013; Gu et al., 2021). Our study reflects the diversity of behaviors of the same animal in the ecological transition zone, confirming the theory of divergent evolution to the same extent (Gulick, 1988; Cao et al., 2011).

### Response of sympatric rodent species to acorn germination

We first showed that sympatric rodent species universally preferred to remove and hoard germinating acorns over nongerminating acorns. Our results showed that rodents with a strong preference for scatter-hoarding and pruning of germinating acorns preferred to consume nongerminating acorns; rodents with both scatter- and larder-hoarding behavior preferred germinating acorns for consumption; and rodents with only larder-hoarding behavior preferred to consume germinating acorns over nongerminating acorns. These results are inconsistent with the perishability hypothesis (Hadj-Chikh et al., 1996) and numerous previous studies showing that rodents selectively disperse and cache dormant seeds over those that have broken dormancy (Steele et al., 2006). One potential explanation for this could be that a large proportion of germinating acorns are pruned, which can be used for long-term storage (Jansen et al., 2006). We also found that diurnal squirrels consume more nongerminating acorns, which is consistent with the previous findings (Yang et al., 2012). A reasonable explanation is that diurnal squirrels are bold, have a large appetite, and prefer to eat acorns *in situ* (31.88% ± 5.63%) than nocturnal rodents (2.56% ± 0.28%). Prioritizing nongerminating acorns maximizes overall storage gains, as pruning radicles and then eating the acorns require significantly more energy than eating intact acorns directly. Consequently, squirrels may have more efficient cache management tactics than nocturnal rodents. In contrast, nocturnal rodents tend to eat more germinating acorns, similar to previous findings (Cao et al., 2011). In particular, rapid consumption of germinating acorns may minimize energy loss for larder hoarders, consistent with the perishability hypothesis (Hadj-Chikh et al., 1996).

### Radicle pruning of sympatric rodent species

Radicle pruning is expected to defectively delay seed germination and be an evolutionary strategy for the cache management of squirrels (Yang et al., 2012; Yi et al., 2013; Steele and Yi, 2020). In this study, we first found that the proportion of radicle pruning of germinating acorns increased as the scatter-hoarding ability of rodents improved. In temperate regions, most WO acorns germinate (radicle elongation) in autumn, but the embryo remains dormant until the following spring. Therefore, radicle pruning seems to be a more efficient food-hoarding strategy during the cold winter. We infer that radicle-pruning behavior may be more widespread in rodents and in a wide range of regions (Jansen et al., 2006; Cao et al., 2011; Yang et al., 2012). Furthermore, our results showed that radicle pruning did not negatively affect seed germination, suggesting that radicle pruning may not serve as an effective strategy for hoarding rodents to counter rapid germination of white oak species (Yang et al., 2012; Zhang et al., 2016; Yi et al., 2021).

### First reports on embryo excision by *A. peninsulae*

Previous studies have suggested that embryo excision is a behavior unique to tree squirrels and that there is a possible co-evolutionary relationship between oaks and squirrels (Vander Wall, 1990; Xiao and Zhang, 2012; Steele and Yi, 2020). We did not find the embryo excision behavior of *S. davidianus* and *T. sibiricus*, although Xiao et al. (2010) reported a high proportion of embryo excision of white oak by *S. davidianus* in a nearby study area. Furthermore, embryo excision by *S. davidianus* has not been reported in other regions. A possible explanation could be that dormant oaks were not distributed in this study area, whereas all squirrels with embryo-excision behavior reported to date have experienced hoarding of both dormant and nondormant acorns in the same space–time environment. Therefore, embryo-excision behavior may be the result of an evolutionary adaptation of squirrels in the long-term food selection of two relatively distinct germination phenotypes (Steele et al., 2006; Steele and Yi, 2020). Interestingly, our observations provide strong evidence for embryo excision behavior in seed-hoarding mice other than squirrels; there are several reasons to believe that this behavior in *A. peninsulae* may be in the early stages of the evolutionary process. First, a lower rate of embryo excision by *A. peninsulae* has been observed compared to that of several tree squirrels (Fox, 1982; Xiao and Zhang, 2012). Second, this behavior is only performed by a limited number of individuals of *A. peninsulae*, whereas studies of tree squirrels have reported that this behavior is generally ubiquitous among conspecifics and is innate and performed by naïve animals without previous experience with acorns (Xiao and Zhang, 2012; Steele and Yi, 2020). Third, it is evident from the germination experiments that embryo excision by *A. peninsulae* is often not deep enough to completely remove the embryo and prevent germination (8% of seedlings survive after embryo excision in this study). Finally, these field mice only performed embryo excision on germinating acorns, suggesting a limited ability to detect seed dormancy. This is the first study to document the behavior of embryo excision as a strategy for long-term cache management of acorns by a rodent species (*A. peninsulae*) other than a squirrel, suggesting a case of convergent evolution possibly in its early stages.

## Conclusions

Although the food-hoarding animals in our study adopted different food-hoarding strategies, they all universally prune the radicles of the germinating acorns before hoarding. Their sensitivity to acorn germination schedules provides a strong evidence for a diffuse co-evolutionary relationship between rodents and oak acorns. There is a strong correlation between radicle pruning behavior and the scatter-hoarding preference of food-hoarding rodents, although radicle pruning by rodents had little effect on seedling development. In addition to radicle pruning, we present the first evidence of embryo excision by a Muridae species (*A. peninsulae*) other than squirrels, which may raise interesting questions concerning the evolution of this adaptive behavior.

## Data availability statement

The raw data supporting the conclusions of this article will be made available by the authors, without undue reservation.

## Ethics statement

The animal study was reviewed and approved by Henan University of Science and Technology.

## Author contributions

XYi conceived and designed the study. MZ, ZD and HC carried out the experiments. MZ, XYa and SL performed data analyses. MZ, XYi and XYa wrote the first draft of the manuscript. All authors contributed to the article and approved the submitted version.
