# Supplementary material for: Behavioral adaptation of sympatric rodents to early germination of oak acorns: radicle pruning and embryo excision
Source: Front Plant Sci. 2023 May 9;14:1135312. doi: 10.3389/fpls.2023.1135312 (PMC10203563; doi:10.3389/fpls.2023.1135312)
Supplement: Supplementary file 1 [file DataSheet_1.docx]

**Supporting information**

Behavioral adaptation of sympatric rodents to early germination of oak acorns: radicle pruning and embryo excision

Mingming Zhang^1^^†^, Xifu Yang^2†^, Zhong Dong^3^, Shuyuan Liu^1^ , Huanhuan Chen^1^ and Xianfeng Yi^1,^ ^4^ *

^1^ College of Agriculture, Henan University of Science and Technology, Luoyang, China.

^2^ State Key Laboratory of Integrated Management of Pest Insects and Rodents, Institute of Zoology, Chinese Academy of Sciences, Beijing, China.

^3^ College of Food and Bioengineering, Henan University of Science and Technology, Luoyang, China.

^4^ School of Life Sciences, Qufu Normal University, Qufu, China.

^†^ These authors have contributed equally to this work.

* **Correspondence:**. E-mail: ympclong@163.com

Table S1 Summary of statistical differences in seed fates (acorn consumption, scatter-hoarding, and radicle pruning) between different rodent species based on generalized linear mixed models in the emmeans package in R software. Fixed factors in bold had significant effects (*P* < 0.05). A.pe – *Apodemus peninsulae*, C.ca – *Cansumys canus*, N.co – *Nivirenter confucianus*, S.da – *Sciurotamias davidianus*, T.si – *Tamias sibiricus*.

| Seed fates | Contrast | Estimate | SD | z | *P* |
| --- | --- | --- | --- | --- | --- |
| Probability of removal | A.pe - C.ca | 0.171 | 0.242 | 0.706 | 0.955 |
|  | **A.pe - N.co** | **0.929** | **0.196** | **4.733** | **<0.001** |
|  | A.pe - S.da | 0.022 | 0.209 | 0.108 | 1.000 |
|  | **A.pe - T.si** | **1.057** | **0.232** | **4.560** | **<0.001** |
|  | **C.ca - N.co** | **0.758** | **0.233** | **3.251** | **0.010** |
|  | C.ca - S.da | -0.149 | 0.221 | -0.672 | 0.963 |
|  | **C.ca - T.si** | **0.885** | **0.243** | **3.645** | **0.002** |
|  | **N.co - S.da** | **-0.907** | **0.199** | **-4.566** | **<0.001** |
|  | N.co - T.si | 0.127 | 0.222 | 0.574 | 0.979 |
|  | **S.da - T.si** | **1.034** | **0.210** | **4.927** | **<0.001** |
| Probability of consumption | A.pe - C.ca | -1.534 | 0.581 | -2.638 | 0.064 |
|  | A.pe - N.co | -0.861 | 0.579 | -1.487 | 0.571 |
|  | **A.pe - S.da** | **-3.173** | **0.487** | **-6.512** | **<0.001** |
|  | **A.pe - T.si** | **-4.110** | **0.497** | **-8.277** | **<0.001** |
|  | C.ca - N.co | 0.672 | 0.509 | 1.321 | 0.678 |
|  | **C.ca - S.da** | **-1.639** | **0.372** | **-4.407** | **<0.001** |
|  | **C.ca - T.si** | **-2.576** | **0.385** | **-6.688** | **<0.001** |
|  | **N.co - S.da** | **-2.311** | **0.399** | **-5.792** | **<0.001** |
|  | **N.co - T.si** | **-3.249** | **0.411** | **-7.903** | **<0.001** |
|  | **S.da - T.si** | **-0.937** | **0.216** | **-4.340** | **<0.001** |
| Probability of scatter-hoarding | **A.pe - N.co** | **1.637** | **0.196** | **8.349** | **<0.001** |
|  | A.pe - S.da | 0.134 | 0.182 | 0.732 | 0.884 |
|  | **A.pe - T.si** | **1.761** | **0.240** | **7.340** | **<0.001** |
|  | **N.co - S.da** | **-1.504** | **0.194** | **-7.759** | **<0.001** |
|  | N.co - T.si | 0.123 | 0.247 | 0.501 | 0.959 |
|  | **S.da - T.si** | **1.627** | **0.223** | **7.291** | **<0.001** |
| Probability of radicle pruning | A.pe - C.ca | 0.473 | 0.261 | 1.813 | 0.366 |
|  | **A.pe - N.co** | **2.256** | **0.362** | **6.236** | **<0.001** |
|  | A.pe - S.da | 0.009 | 0.199 | 0.045 | 1.000 |
|  | A.pe - T.si | 0.339 | 0.254 | 1.337 | 0.668 |
|  | **C.ca - N.co** | **1.783** | **0.408** | **4.365** | **<0.001** |
|  | C.ca - S.da | -0.464 | 0.253 | -1.835 | 0.353 |
|  | C.ca - T.si | -0.134 | 0.298 | -0.449 | 0.992 |
|  | **N.co - S.da** | **-2.247** | **0.372** | **-6.044** | **<0.001** |
|  | **N.co - T.si** | **-1.917** | **0.404** | **-4.747** | **<0.001** |
|  | S.da - T.si | 0.330 | 0.246 | 1.346 | 0.663 |

Table S2 Summary of statistical differences in seed fates (acorn removal, consumption, and scatter-hoarding) between germinating and non-germinating *Quercus variabilis* acorns by different rodent species based on generalized linear mixed models in the lme4 package of R software. Fixed factors in bold had significant effects (*P* < 0.05).

| Seed fates | Rodent species | | Estimate | Std. Error | *z* | *P* |
| --- | --- | --- | --- | --- | --- | --- |
| Probability of removal | *A. peninsulae* | (Intercept) | 3.465 | 0.663 | 5.229 | <0.001 |
|  |  | **Non-germinating** | **-1.464** | **0.330** | **-4.432** | **<0.001** |
|  | *C. canus* | (Intercept) | 2.962 | 1.722 | 1.720 | 0.085 |
|  |  | Non-germinating | 0.218 | 0.571 | 0.381 | 0.703 |
|  | *N. confucianus* | (Intercept) | 0.935 | 0.634 | 1.476 | 0.140 |
|  |  | Non-germinating | -0.273 | 0.321 | -0.848 | 0.396 |
|  | *S. davidianus* | (Intercept) | 1.496 | 0.551 | 2.716 | 0.007 |
|  |  | Non-germinating | -0.326 | 0.302 | -1.080 | 0.280 |
|  | *T. sibiricus* | (Intercept) | 0.056 | 0.296 | 0.188 | 0.851 |
|  |  | Non-germinating | 0.053 | 0.326 | 0.163 | 0.870 |
| Probability of consumption | *A. peninsulae* | (Intercept) | -1.410 | 0.214 | -6.579 | <0.001 |
|  |  | Non-germinating | -0.259 | 0.253 | -1.021 | 0.307 |
|  | *C. canus* | (Intercept) | 9.406 | 3.591 | 2.619 | 0.009 |
|  |  | **Non-germinating** | **-1.464** | **0.605** | **-2.420** | **0.016** |
|  | *N. confucianus* | (Intercept) | -0.634 | 0.453 | -1.399 | 0.162 |
|  |  | Non-germinating | -0.211 | 0.287 | -0.736 | 0.462 |
|  | *S. davidianus* | (Intercept) | -0.861 | 0.430 | -2.001 | 0.045 |
|  |  | **Non-germinating** | **0.536** | **0.260** | **2.063** | **0.039** |
|  | *T. sibiricus* | (Intercept) | 0.161 | 0.292 | 0.550 | 0.582 |
|  |  | **Non-germinating** | **1.383** | **0.376** | **3.675** | **<0.001** |
| Probability of scatter-hoarding | *A. peninsulae* | (Intercept) | 0.964 | 0.306 | 3.149 | 0.002 |
|  |  | **Non-germinating** | **-0.578** | **0.230** | **-2.518** | **0.012** |
|  | *C. canus* | (Intercept) | - | - | - | - |
|  |  | Non-germinating | - | - | - | - |
|  | *N. confucianus* | (Intercept) | -1.359 | 0.003 | -423.497 | <0.001 |
|  |  | Non-germinating | -0.002 | 0.003 | -0.642 | 0.521 |
|  | *S. davidianus* | (Intercept) | 0.690 | 0.492 | 1.401 | 0.161 |
|  |  | **Non-germinating** | **-0.698** | **0.260** | **-2.679** | **0.007** |
|  | *T. sibiricus* | (Intercept) | -0.610 | 0.323 | -1.888 | 0.059 |
|  |  | **Non-germinating** | **-1.248** | **0.404** | **-3.086** | **0.002** |

Table S3 Summary of statistical differences in germination rate of embryo excision and radicle pruning *Quercus variabilis* acorns by *Apodemus peninsulae* based on the *Chisq.test* function in the stats package of R software. Fixed factors in bold had significant effects (*P* < 0.05).

| Contrast | χ^2^ | df | *P* |
| --- | --- | --- | --- |
| Embryo excision – Radicle pruning | 0.936 | 1 | 0.334 |
| **Embryo excision** – **Intact** | **51.532** | **1** | **< 0.001** |
| **Radicle pruning** – **Intact** | **32.204** | **1** | **< 0.001** |


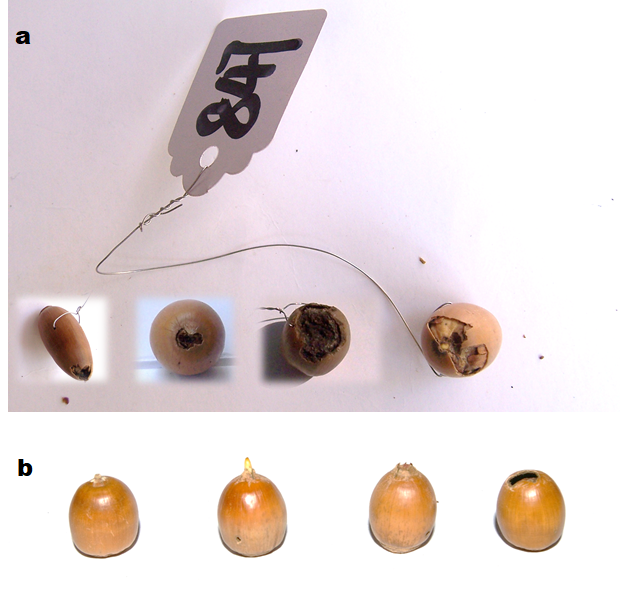


Fig. S1 (a) Embryo excision of *Quercus variabilis* acorns by *Apodemus peninsulae* in enclosure experiment; (b) the acron status of *Q. variabilis*: non-germinating, germinating, radical pruning, embryo excision (from left to right).
